# Lineage-specific regulatory evolution: insights from massively parallel reporter assays

**DOI:** 10.1016/j.gde.2025.102372

**Published:** 2025-06-20

**Authors:** Ryder Easterlin, Nadav Ahituv

**Affiliations:** 1 Department of Bioengineering and Therapeutic Sciences, University of California San Francisco, San Francisco, CA, USA; 2 Institute for Human Genetics, University of California San Francisco, San Francisco, CA, USA

## Abstract

Lineage-specific genetic variants play a key role in evolutionary divergence, particularly through changes in *cis*-regulatory elements that fine-tune gene expression. Massively parallel reporter assays (MPRAs) provide a powerful approach to characterize these variants at scale. This review highlights how MPRAs have been used to study lineage-specific regulatory activity in enhancer elements, including human accelerated regions, human adaptive quickly evolving regions, and short human-specific conserved deletions. We discuss the effects of enhancer variation on traits distinguishing modern humans, archaic hominins, and primates, as well as how MPRAs disentangle *cis*- and *trans*-regulatory contributions to gene expression divergence. As MPRA technology advances, integrating it with CRISPR-based validation and artificial intelligence–driven predictions will further illuminate the role of lineage-specific regulatory evolution.

## Introduction

The study of evolution and diversity has rapidly progressed into the genomics era with the advent of steadily more accessible high-throughput technologies and an increasing breadth of whole-genome sequencing of various extinct and extant lineages [1–3]. This brings us within striking distance of a longstanding question in evolution and biology — deciphering exactly which genetic variants are responsible for certain phenotypic differences between closely related lineages, and how they influence underlying biology from the cellular to the organismal scale. In this review, we focus primarily on primate evolution, as there has been particular emphasis recently in the evolutionary functional genomics space on identifying genetic changes in putative enhancer elements that distinguish modern humans from other primate and human lineages.

In particular, variants and sequences only present in a certain evolutionary lineage are a primary driver of unique, lineage-specific adaptation and divergence [4]. For example, a modern human-specific nonsynonymous variant in the *NOVA1* coding region was indicated to have a role in humans’ unique capacity for vocal learning and communication [5]. While protein-coding variants such as the *NOVA1* mutation can have a high effect size, their relative scarcity between closely related lineages suggests that the noncoding genome plays a larger role in the divergence of close evolutionary neighbors [6,7]. Variations between species often occur within *cis*-regulatory elements (CREs) — such as enhancers and promoters — that drive cell type–specific transcriptional programs, facilitating evolutionary fine-tuning of gene expression [8,9]. Variation in these CREs can impact when, where, and to what extent genes are expressed, thereby influencing the functional evolution of certain cell types across species while minimizing pleiotropic effects that may have more drastic effects on fitness [10]. Lineage-specific variants, whether acting independently or together in the same CRE, have been shown to play a significant role in driving lineage-specific adaptations and traits [4]. Such lineage-specific regulatory changes are often linked to traits that are critical for success in distinct environments, such as the unique neurodevelopmental adaptations in humans that underpin advanced cognition [11–13].

The same biological complexities of gene regulation still present challenges to research. While we know that the majority of trait-associated, functional genetic variation occurs in noncoding regions of the genome, the consequences of these variants are not as readily accessible as those in protein-coding regions. The interplay of multiple genetic elements, epigenetic modifications, and environmental influences complicates efforts to directly link specific variants to functional outcomes [14]. In addition, the context-dependent nature of CREs means that the effects of variants may vary across different tissues, developmental stages, and evolutionary lineages [15]. Thus, identifying exactly which genetic changes are causal for phenotypic divergence between closely or distantly related lineages largely remains an unsurmounted challenge.

Massively parallel reporter assays (MPRAs) can address these limitations by testing the functional activity of hundreds of thousands of sequences in a single experiment [16,17]. A widely used version of MPRA places a candidate CRE in front of a minimal promoter and transcribed barcode, allowing regulatory activity to be assayed by measuring the RNA levels of the transcribed barcode. First developed as an episomal assay [18], the design has been adapted to a lentiviral delivery format (lentiMPRA) [19], expanding the range of accessible cell types and enabling transcriptional readouts of tested CREs from an integrated genomic context. A side-by-side comparison of the same MPRA library in an episomal and lentivirus integrated manner showed that lentiMPRA provides higher correlation with ENCODE regulatory annotations, cell-type predictions, and sequence-based models [20]. However, both episomal and lentiviral MPRA designs measure regulatory activity outside the native genomic locus and therefore do not perfectly capture *in vivo* regulatory dynamics [21].

A related approach, Self-Transcribing Active Regulatory Region sequencing (STARR-seq) [22], has also been used to study gene regulation in the context of evolution [23–25]. It differs from typical MPRA designs by placing candidate regulatory sequences downstream of a minimal promoter so that each element serves as its own barcode. This allows direct quantification of enhancer activity based on the abundance of self-transcribed RNA. Together, MPRA and STARR-seq designs (which we will refer to together as MPRA for brevity) have dramatically expanded the scale and resolution at which regulatory function can be investigated, enabling high-throughput functional testing of essentially any DNA sequence. One of the most common uses of MPRA tests the effect that genetic variants in promoters and enhancers have on *cis*-regulation [26]. Often used to study disease-associated variants in noncoding regions, this type of design is now of particular relevance to evolutionary fields, as we can now test the cell type–specific gene regulatory effect of tens of thousands of lineage-specific variants and sequences.

**Figure 1:**
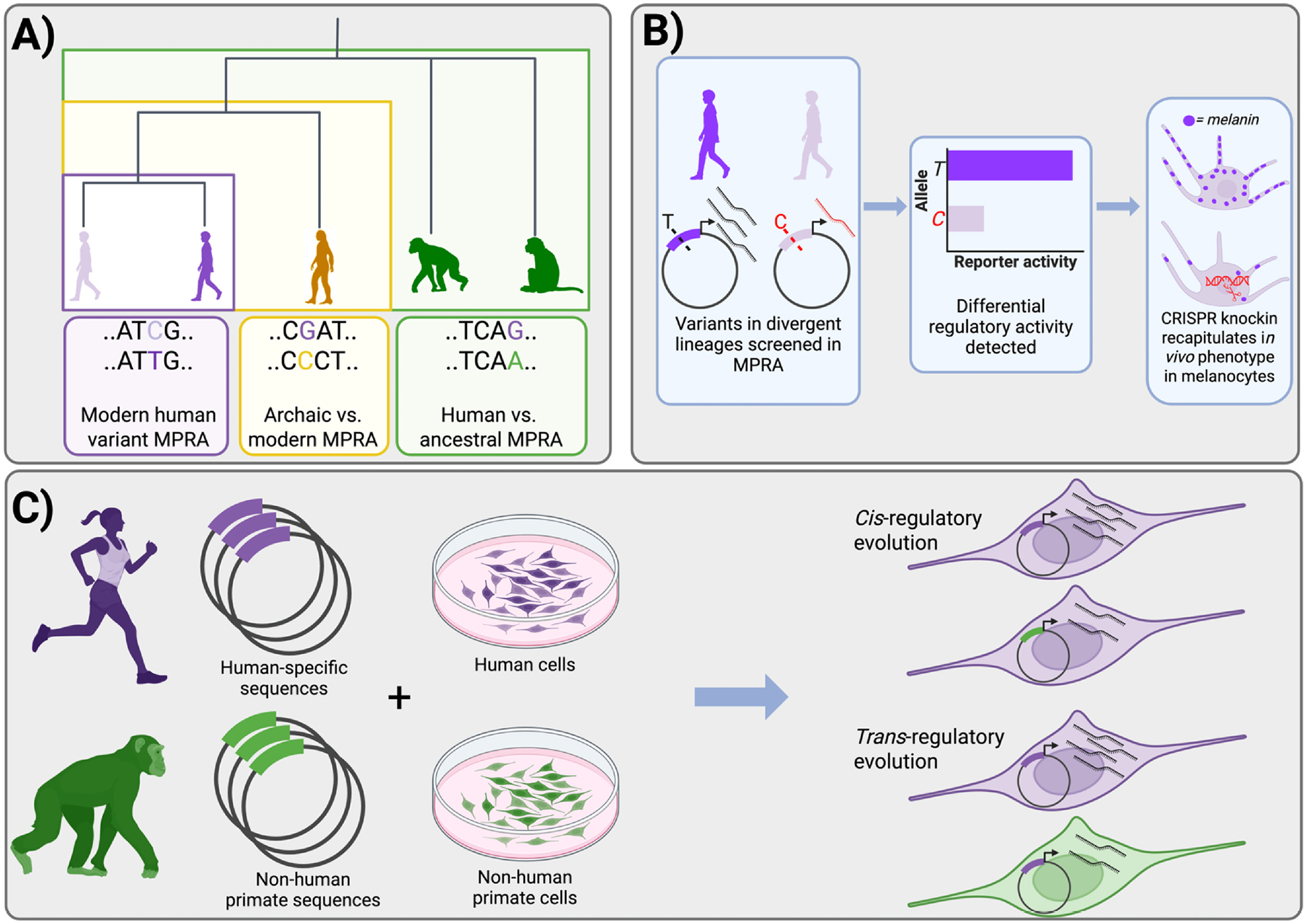
Lineage-specific MPRAs: Identifying regulatory divergence and *cis-trans* effects on gene regulation. **(a)** Lineage-specific MPRAs have focused on characterizing human lineage–specific variants, either between modern human lineages, modern and archaic humans, or humans and non-human primates. **(b)** Lineage-specific mutations and sequences that drive differential gene regulation can be discovered with lineage-specific MPRAs, nominating these candidates for further validation via models derived with CRISPR genome editing. **(c)** *Cis* versus *trans* effects can be determined with lineage-specific MPRAs when both lineages’ sequences are assayed in cells derived from each lineage.

This review explores how MPRA has been used to understand the functional impact of lineage-specific genetic variants and sequences. In particular, we highlight recent studies that directly compare the gene regulatory effects of alleles and sequences that are almost or completely fixed in one lineage and absent in another. Recently, these studies have predominantly focused on variants that distinguish humans from other lineages across multiple timescales, including comparisons between humans and chimpanzees or other primates [27], modern humans and archaic human groups [28], and finally between different modern human populations [29,30] (Figure 1a). We will focus on two primary categories of findings: first, the identification and characterization of lineage-specific variants and sequences that drive distinct traits between lineages (Figure 1b), and second, evaluating the relative contributions of *cis*-regulatory versus *trans*-regulatory effects in the evolution of gene expression (Figure 1c).

## Massively parallel reporter assays for characterization

Two previous studies have focused on assessing the regulatory impact of human-derived variants in human accelerated regions (HARs) [31] and human adaptive quickly evolving regions (HAQERs) [24]. HARs, first described in 2006 [32], are regions strongly conserved in vertebrates that have rapidly accumulated variants in the human lineage. HAQERs are regions harboring accelerated human-specific mutations emerging from more neutral areas of the genome. An MPRA and single-cell STARR-seq experiment, respectively assessing both types of regions, found significant overlaps of human-specific sequences in enhancers active in neurodevelopment [24,33]. Another MPRA conducted on 14,042 modern human-specific variants genome-wide found an enrichment of differentially active genes involved in cerebellum and vocal tract development [12]. Finally, transposable elements (TEs) comprise a substantial portion of mammalian genomes and have been increasingly studied as a source of lineage-specific regulatory elements [34]. Notably, TE-derived regulatory elements are largely species-specific since the human–mouse divergence [34], and other studies estimate that they constitute a significant fraction of all lineage-specific regulatory elements [35]. A recent MPRA investigating primate-derived regulatory elements within the LTR18A subfamily revealed the cell type–specific regulatory activity of TE-derived sequences in human cells [36], highlighting their potential role in shaping species and tissue-specific gene expression programs. It is important to note that MPRAs used in these studies generally follow one of two strategies. Certain studies [13,24,25,37] test full orthologous or derived sequences to measure net regulatory effects, and others introduce individual variants into a common background to isolate the impact of specific mutations [12,33,38]. While the former captures cumulative effects, including epistasis, the latter allows finer resolution of changes introduced by specific variants.

More recently, Xue et al. identified short human-specific conserved deletions (hCONDELS) [39] in otherwise deeply conserved regions and used an episomal MPRA to test their effect on *cis*-regulation in six human cell types, including neural progenitor cells [13]. They found 800 hCONDELs that altered regulatory activity, with one-third driving cell type–specific effects. Notably, 42% of these deletions increased activity, and more than half disrupted transcription factor binding, suggesting that loss of repressive motifs can be a mode of evolution that enhances *cis*-regulatory function. This MPRA also pinpointed hCONDELs affecting human-specific expression of the neurodevelopmental genes *PPP2CA* and *LOXL2*, which were validated to affect gene expression via CRISPR editing.

A growing use of MPRA is to identify phenotypic effects of variants separating younger lineages, including regional human populations. Previous studies have often focused on the effects of archaic-introgressed variants that are often positively selected in only certain lineages [40–44]. In a recent study, Feng et al. [38] conducted an MPRA in melanocytes to identify single-nucleotide polymorphisms (SNPs) influencing skin pigmentation in present-day Africans with diverse local ancestries. One variant near *OCA2*, a gene linked to human pigmentation [45], conferred strong differential regulatory activity compared to the reference allele and was shown to affect *OCA2* expression and melanin production in CRISPR-edited melanocytes.

Together, these studies demonstrate how MPRAs can be leveraged to identify regulatory variants with functional consequences across both ancient and more recent evolutionary timescales. However, while identifying lineage-specific regulatory changes driven by sequence divergence (*cis* effects) is informative, it captures only part of the picture. Total regulatory divergence between lineages results from the combined influence of both *cis* and *trans* effect differences in the cellular or molecular environment that modulate how regulatory sequences are interpreted [46]. Addressing this distinction is essential for understanding the mechanisms driving regulatory evolution.

## Massively parallel reporter assays to deconvolute *cis* versus *trans* effects in lineage divergence

To that end, there has been an increasing number of MPRAs to deconvolute the effects of *cis* changes versus *trans* effects on gene expression divergence. At the surface, *cis* changes affect transcription factor binding, while separate *trans* environments can have different transcription factor availability [47,48]. Both mechanisms can cause divergent gene regulatory activity of an element. MPRAs can determine whether *cis, trans*, or both effects are predominant by testing two sequence versions from different lineages in the cellular contexts of both species. Mattioli et al. used MPRAs in human and mouse embryonic stem cells to separate *cis* and *trans* contributions to regulatory divergence [49]. They found that *cis* effects were common in both promoters and enhancers, while *trans* effects were less frequent and more prominent in enhancers. Promoters often showed compensatory *cis* and *trans* changes to maintain stable expression, whereas enhancers balanced activity across multiple elements targeting the same gene. This study provided functional evidence for the distinct contributions of *cis* and *trans* effects, highlighting that different regulatory elements evolve through different mechanisms. It also emphasized the need to assess these contributions across a broader range of cell types.

As part of a systematic dissection of the functional effects of human-derived variants in HARs, Whalen et al. [37] conducted a lentiMPRA to compare the enhancer activity of chimpanzee and human versions of 714 HARs. HARs have been studied in depth across a variety of experimental and bioinformatics modalities, and have been indicated to be functionally linked to the divergence of human brain development and cognition from other vertebrates, including chimpanzees, our closest extant relative [11]. Sequences covering 714 HARs were introduced into chimpanzee and human iPSC-derived neural progenitors to separate *trans*-regulatory differences from sequence change-driven activity divergence. They found that for 293 active HARs, whether the cellular context was human or chimp had little differentiating effect for a given sequence version. However, when humans and chimpanzees are compared in a single *trans* environment, more than half (54%) of the active HARs exhibited sequence change-dependent differential activity. This suggests that for these rapidly evolved elements, sequence changes play a dominant role in driving lineage-specific enhancer activity, rather than differences in the *trans* environment. Similarly, a study using human, chimp, and hybrid embryoid bodies found that *cis*-regulatory changes, while actually less common than *trans*-regulatory changes genome-wide, had larger effect sizes on gene expression [50]. Moreover, genes with greater *cis*-regulatory divergence were associated with higher coding sequence substitution rates and overall sequence divergence, suggesting that genes under less coding constraint are more susceptible to evolutionary shifts in their regulatory elements [50].

In another study comparing human regulatory elements to those of another primate in both species’ cellular contexts, Hansen et al. [25] find *cis* and *trans* effects have similar contributions to activity differences between human and rhesus macaque. Here, chromatin-accessible DNA from human and macaque lymphoblastoid cells was used in a STARR-seq experiment (ATAC-STARR-seq) in both species’ cells. Importantly, this approach enabled the capture of a broad and unbiased landscape of accessible regulatory elements in both species, rather than relying on predefined design choices based on human data that may miss active elements in other, less investigated species. In their study, the authors found that most (67%) of the elements active in only one species are sensitive to both the *cis* and *trans* differences between human and macaque. They additionally report that there are about as many sequences in both species that are sensitive to *cis*-only changes as there are to *trans*-only changes.

These two studies measure different relative contributions of *cis* versus *trans* effects, likely due to differences in the sequences tested. First, HARs underwent rapid sequence evolution only in humans, with many of these changes likely selected because of their strong impact on *cis*-regulation [32]. On the contrary, genome-wide accessible sequences that were tested in the ATAC-STARR-seq study are likely under less selective pressure on average than HARs. It is possible that the longer evolutionary time separating humans and macaque — ~25 million years, to the ~6 million years separating human and chimp — leads to greater divergence in their *trans*-regulatory networks. That said, it is generally considered that evolution in *cis*-regulation is more stable, and *trans* effects may actually get buffered over time [51,52]. Finally, different cell types — such as neural versus immune, as outlined here — and biological pathways likely evolve under distinct selective pressures, leading to variation in the relative contributions of *cis*- and *trans*-regulatory evolution [10,47,50]. The question of *cis* and *trans*-acting evolution is being extensively studied across systems, and MPRAs can be a primary mode to query these effects at the level of CRE evolution across many cell types.

## Discussion

Evolutionary adaptation and divergence are shaped by fine-tuning CREs. Variants and sequences with polarized allele frequencies between populations or species can drive significant differences in regulatory activity, influencing key traits and contributing to the divergence of phenotypes [53,54]. These variants typically exhibit larger effect sizes than common variants within populations, as they are frequently subject to stronger selective pressures. Understanding the functional impacts of these variants also opens the door to exploring how lineage-specific regulatory changes not only contribute to phenotypic diversity but may also offer new therapeutic modalities by mimicking or harnessing evolutionary adaptations [54]. By studying these lineage-specific adaptations and their genetic precursors, we can better comprehend the molecular mechanisms underlying adaptation and potentially apply this knowledge to gene therapy and precision medicine.

To investigate these noncoding variations functionally, MPRAs have emerged as a powerful tool, complementing CRISPR-based approaches for screening regulatory elements [55–61]. MPRAs offer certain technical and conceptual advantages: they can test thousands of precisely designed sequences in parallel without the need for endogenous editing, enabling broader and deeper exploration of regulatory sequence space [16]. CRISPR-based perturbations, like CRISPRi screens, often require more extensive optimization and may affect larger genomic regions than the targeted element, complicating the interpretation of *cis*-regulatory effects [58]. At the same time, MPRA also has inherent limitations: short synthetic fragments centered on specific variants can isolate individual effects but often miss critical distal interactions and broader regulatory and cellular context. Moreover, MPRAs do not directly assess phenotypic consequences of genetic variation. In contrast, perturbing endogenous loci using CRISPR-based technologies can capture compensatory effects across multiple regulatory elements and provide a more direct model for studying gene regulation and phenotype, features inherently absent in the ectopic context of MPRA.

Ultimately, the value of both approaches depends on selecting developmentally and physiologically relevant cell types, as well as assaying regulatory regions active in those contexts to appropriately address the evolutionary question at hand. Looking forward, we project an increasing use of MPRA to deeply query individual regulatory elements in evolutionary studies, building on approaches like saturation mutagenesis [62] and locus tiling [63] that have been more common in studies that dissect disease-relevant elements. These approaches can enable a high-resolution view of how certain variant combinations can exact nonlinear or compensatory effects on individual elements that are missed by MPRAs querying the effect of variants individually. For evolutionary questions, MPRAs inspired by these methods could enable comparison of ancestral and derived versions of regulatory loci from multiple related lineages [36], offering an approach to reconstruct the temporal sequence of mutational events leading to lineage-specific regulatory activity in critical cell types.

Even with its growing utility, another limitation of MPRA continues to be its reliance on cell culture systems, which do not fully recapitulate the complex regulatory landscape of the tissue of origin [16]. Many MPRAs are performed in immortalized cell lines, which often diverge from their primary counterparts in terms of chromatin accessibility, transcription factor abundance, and epigenetic modifications, all of which can influence regulatory element activity. Notably, differences in regulatory networks, even between primary cell lines and tissues, have been documented [64], suggesting that results obtained from MPRAs *in vitro* may not always translate *in vivo*.

To bridge this gap, recent work showed the ability to deliver MPRA constructs to numerous tissues in adult mouse using retro-orbital injection of an adeno-associated virus (AAV)-based library [65]. Other studies have had success in using AAV systems to perform MPRAs in the eye and brain [66,67]. However, scaled delivery of large MPRA libraries to tissues *in vivo* remains a major challenge. To date, this approach has been largely limited to testing sequences in adult tissues, and recent studies have identified issues, such as chimerism, when using AAV-based delivery systems, which can complicate the interpretation of regulatory activity [68]. Further development of MPRA such that it can consistently work in embryos and multiple tissues will be extremely beneficial, though with the caveat that these assays will most likely be infeasible in most species beyond certain model organisms. Finally, single-cell MPRA [69–71] is another promising direction, offering the potential to resolve regulatory activity at cell-type resolution within heterogeneous populations, though at a more limited scope of candidate variants than traditional MPRAs. As MPRA technologies continue to evolve, parallel advances in aforementioned CRISPR-based assays, which can test the effects of thousands of variants in a single experiment in cells [58–61], and eventually in whole organisms, could further accelerate discovery. Together, we expect these tools will more efficiently uncover the phenotypic consequences of evolutionary variants, especially as new assays expand the range of measurable outputs beyond gene expression and viability.

Another current development is artificial intelligence (AI). Developments in machine learning will also rapidly accelerate each part of the identification, characterization, and validation pipeline outlined here. Already, Whalen et al. [37] utilized a basic linear model to distinguish between epigenetic profiles of brain enhancers versus enhancers active in other cell types to prioritize HARs for MPRA and queried the effects of human-specific mutations on chromatin state using the deep learning model Sei [72]. The ongoing improvement of models that can learn the grammar of cell type–specific regulatory activity directly from sequence will be even more powerful for the purpose of variant effect prediction [73–75]. However, current DNA sequence-based models remain limited in their ability to accurately predict the regulatory effects of genetic variants within human populations [76], and this limitation needs to be addressed before such models can be reliably extended to interpret variant effects across species where distinct cellular and regulatory contexts further complicate prediction. Additionally, models designed to detect regions that have undergone selective sweeps in evolutionary lineages will be further refined with advances in model architecture [77,78] and the increasing availability of whole-genome and SNP-based sequencing data [29,79]. These advancements in AI and expanding databases of genetic variation toolkits will be pivotal in prioritizing functional lineage-specific variants, and we anticipate that they will go hand-in-hand with experimental assays in investigating the *cis*-regulatory basis of lineage-specific adaptation.

## Data Availability

No data were used for the research described in the article.
